# Metabolic Imaging and Biological Assessment: Platforms to Evaluate Acute Lung Injury and Inflammation

**DOI:** 10.3389/fphys.2020.00937

**Published:** 2020-08-31

**Authors:** Mehrdad Pourfathi, Stephen J. Kadlecek, Shampa Chatterjee, Rahim R. Rizi

**Affiliations:** ^1^Department of Radiology, University of Pennsylvania, Philadelphia, PA, United States; ^2^Department of Physiology, University of Pennsylvania, Philadelphia, PA, United States

**Keywords:** lung inflammation, lung injury, ARDS, FDG-PET, HP-MRI

## Abstract

Pulmonary inflammation is a hallmark of several pulmonary disorders including acute lung injury and acute respiratory distress syndrome. Moreover, it has been shown that patients with hyperinflammatory phenotype have a significantly higher mortality rate. Despite this, current therapeutic approaches focus on managing the injury rather than subsiding the inflammatory burden of the lung. This is because of the lack of appropriate non-invasive biomarkers that can be used clinically to assess pulmonary inflammation. In this review, we discuss two metabolic imaging tools that can be used to non-invasively assess lung inflammation. The first method, Positron Emission Tomography (PET), is widely used in clinical oncology and quantifies flux in metabolic pathways by measuring uptake of a radiolabeled molecule into the cells. The second method, hyperpolarized ^13^C MRI, is an emerging tool that interrogates the branching points of the metabolic pathways to quantify the fate of metabolites. We discuss the differences and similarities between these techniques and discuss their clinical applications.

## Introduction

Acute respiratory distress syndrome (ARDS) and acute lung injury (ALI) are an acute conditions characterized by pulmonary infiltrates (visible in a chest radiograph) arising from pulmonary inflammation, decreased lung compliance, increased vascular permeability and edema (Ards Definition Task Force Ranieri et al., 2012; Pham and Rubenfeld, 2017). Approximately 200,000 patients each year in the US are diagnosed with ALI/ARDS, of which ~10% of patients admitted to the intensive care unit (ICU) (Johnson and Matthay, 2010; Ards Definition Task Force Ranieri et al., 2012). Despite being defined over fifty years ago (Ashbaugh et al., 2005), ARDS remains a significant source of mortality in critically ill patients (Steinberg et al., 1994; Miller et al., 1996).

Respiratory failure from ARDS secondary to coronavirus disease 2019 (COVID-19) is a significant clinical challenge. What is more is that the number of patients and mortalities are anticipated to significantly rise in immediate future (Gattinoni et al., 2020; Ramanathan et al., 2020). As such, it is timely to focus our attention toward acute lung injury and tools that can provide additional insight into the biological mechanisms of ARDS and lung inflammation. Such techniques may not only enable earlier detection of lung injury but also can facilitate more effective strategies to monitor patient’s response to maneuvers and pharmacological interventions. The latter is crucial especially as identifying optimal interventions may alleviate the numerous difficulties of ARDS survivors including physical and psychological sequelae, exercise limitation, and increased use of health care services (Herridge, 2017; Thompson et al., 2017).

Pulmonary inflammation has been implicated in the pathogenesis and progression of ARDS and ALI. An important feature of lung inflammation, is the pivotal role of the vascular endothelium in the onset and amplification of inflammation. The endothelial layer is, by virtue of its location, an interface between flowing blood and the tissue and is thus a converging site of inflammation whereby immune cells adhere to the vessel wall, followed by their transmigration into tissue.

The severity of inflammation and cellular changes during early stages of ARDS, is shown to be predictive of progression of injury and eventual outcome (Miller et al., 1996; Shankar-Hari and McAuley, 2017). Indeed ~33% of ARDS patients have a “hyper-inflammatory” sub-phenotype with a significantly higher mortality rate (Shankar-Hari and McAuley, 2017). Currently ARDS is managed in the ICU through careful optimization of mechanical ventilation to protect the lungs from ventilator-induced injury (Johnson and Matthay, 2010; Fanelli and Ranieri, 2015; Cereda et al., 2016; Tabuchi et al., 2016). However, these protocols do not limit the spread of inflammation (Johnson and Matthay, 2010; Fanelli and Ranieri, 2015).

Currently assessment of pulmonary dysfunction primarily relies on global functional parameters, such as pulmonary function test (PFT) or anatomical imaging tools. These do not provide cellular or molecular information. Histological and biological assessment of the tissue or bronchoalveolar lavage can provide information on inflammatory and injury biomarkers in lung tissue but do not provide regional information and cannot be used for longitudinal monitoring of the severity of lung inflammation and disease. Therefore, tools that enable early detection of pulmonary inflammation followed by its longitudinal assessment can help physicians identify ARDS patients with hyperinflammatory phenotype and monitor their response to pharmacological interventions to select a therapeutic strategy that works best for those patients to ultimately improve outcome.

**FIGURE 1:**
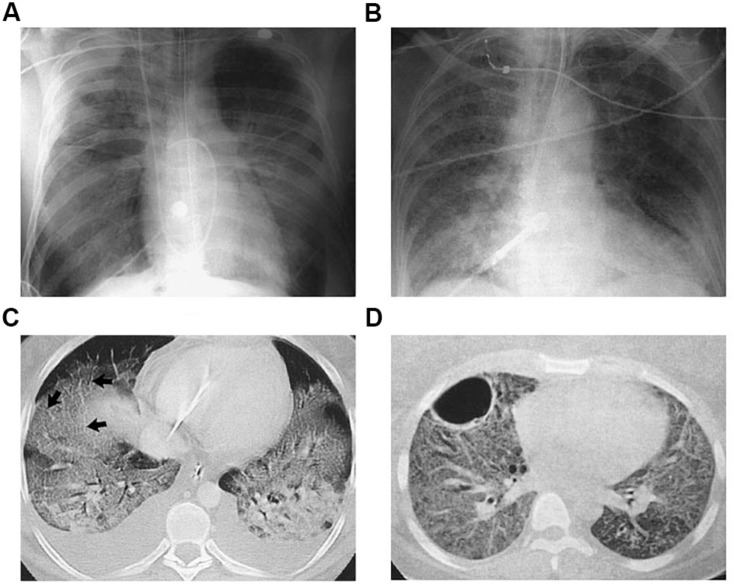
Radiographic and Computed Tomographic (CT) Findings in the Acute, or Exudative, Phase (Panels **A,C**) and the Fibrosing-Alveolitis Phase (Panels **B,D**) of Acute Lung Injury and the Acute Respiratory Distress Syndrome (ARDS). Panel **(A)** shows a chest radiograph from a patient with the ARDS associated with gram-negative sepsis who was receiving mechanical ventilation. There are diffuse bilateral alveolar opacities consistent with the presence of pulmonary edema. Panel **(B)** shows an anteroposterior chest radiograph from another patient with ARDS who had been receiving mechanical ventilation for seven days. Reticular opacities are present throughout both lung fields, a finding suggestive of the development of fibrosing alveolitis. Panel **(C)** shows a CT scan of the chest obtained during the acute phase. The bilateral alveolar opacities are denser in the dependent, posterior lung zones, with sparing of the anterior lung fields. The arrows indicate thickened interlobular septa, consistent with the presence of pulmonary edema. Panel **(D)** shows a CT scan of the chest obtained during the fibrosing-alveolitis phase. There are reticular opacities and diffuse ground-glass opacities throughout both lung fields, and a large bulla is present in the left anterior hemithorax. Reproduced with permission from Ware and Matthay (2000).

There are a number of imaging modalities used for diagnosis and clinical management of ARDS (Mills, 2003; Mistry et al., 2008; Cereda et al., 2016; Thompson et al., 2017). Chest X-ray radiography and computed tomography (CT) are extensively used imaging techniques to assess lung inflammation, injury and progression, where edema and immune cell infiltrates appear as opacities (Figure 1). However it is not trivial to distinguish opacities caused by infiltration, edema, or atelectasis (Cereda et al., 2019). Moreover, these approaches focus on examining the secondary effects of inflammation rather than directly targeting the metabolic processes that are the underlying drivers of inflammation-induced change.

Molecular imaging tools enable non-invasive interrogation of lung cellularity, and therefore can assess inflammatory activity providing critical information about disease progression, response to therapy and prognosis in real time. The purpose of this review article is to provide a brief overview of two state-of-the-art molecular imaging techniques that are currently used pre-clinically. Both methods exploit alterations in lung metabolism as a result of inflammation to visualize regions with active inflammation. These non-invasive molecular imaging techniques can be used as novel platforms to evaluate pulmonary signaling associated with ARDS/ALI and be integrated with functional and physiological parameters obtained by from patients to improve patient prognosis and outcome.

The two state of art molecular imaging discussed here are Positron Emission Tomography (PET) and Hyperpolarized ^13^C Magnetic Resonance Spectroscopic Imaging (HP ^13^C-MRSI), a method recently developed that provides similar information to PET imaging using MRI.

## Pathogenesis of ARDS and Pulmonary Inflammation

**FIGURE 2:**
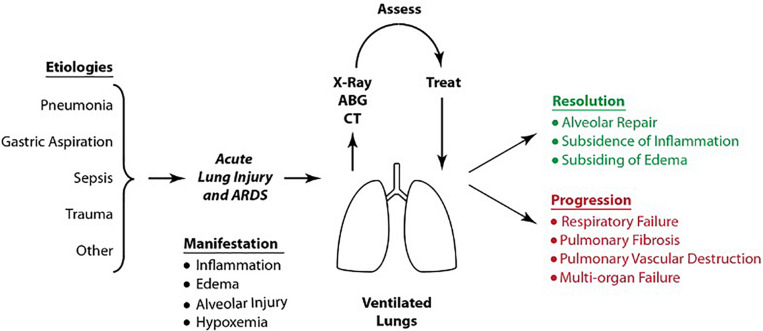
Etiologies, manifestations and sequelae of ARDS/ALI. Upon diagnosis, patients undergo supportive therapy using protective ventilation. The patient’s overall condition is assessed by chest radiography, computed tomography, ventilatory parameters and ABG. Treatment either results in resolution of ARDS/ALI or in the syndrome’s progression into severe respiratory failure, pulmonary fibrosis and eventual multi-organ failure. Reproduced with permission from Pourfathi (2019).

Acute respiratory distress syndrome and acute lung injury (ARDS/ALI) is characterized by sudden onset of respiratory failure, pulmonary edema, diffused alveolar damage and widespread inflammation of the lung (Thompson et al., 2017). There are two pathogenetic pathways leading to ARDS; pulmonary and extra pulmonary ARDS (Pelosi et al., 2003). In pulmonary ARDS, primary lungs injury occurs by a direct insult (e.g., pneumonia, gastric aspiration, or toxin inhalation). In extrapulmonary ARDS, widespread inflammation occurs as a result of a systemic injury (e.g., sepsis, burn injury, or cardiopulmonary bypass), which results in secondary lung injury. In both scenarios, lung injury may progress to ARDS, as shown in Figure 2. Such progression is followed by resolution of ARDS/ALI in response to treatment or further progression into severe respiratory and multi-organ failure.

**FIGURE 3:**
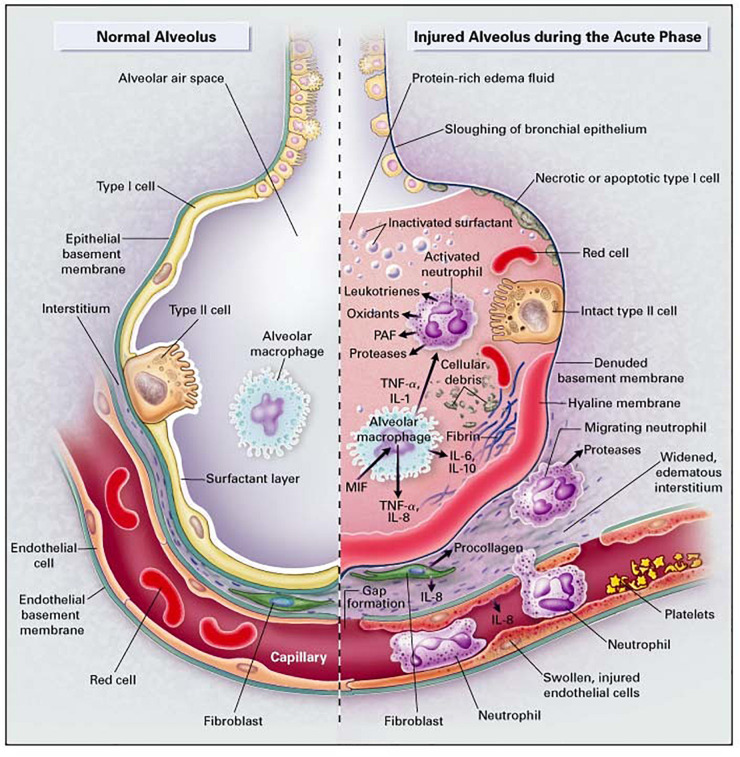
The healthy lung (left), and the acute phase of ARDS/ALI (right). In ARDS/ALI, injury is initiated by either direct or indirect insults to the delicate alveolar structure of the distal lung and associated micro-vasculature. In the acute phase of the injury, resident alveolar macrophages are activated, leading to the release of potent pro-inflammatory mediators and chemokines that promote the accumulation of neutrophils and monocytes. Reproduced with permission from Ware and Matthay (2000).

In both types of ARDS, the endothelium plays a key role in the onset of inflammation. In pulmonary ARDS, local alveolar inflammatory response affects the alveolar endothelium. On the other hand, in systemic ARDS, the inflammatory mediators present in the bloodstream damage the microvascular endothelium. Subsequently, the alveolar or the microvascular endothelium layer is activated, which leads lead to production of cellular adhesion molecule (CAM) and proinflammatory cytokines. The elevation of inflammatory mediators leads to recruitment and adherence of polymorphonuclear neutrophils. In the acute, or exudative, phase of ARDS/ALI (Figure 3), the alveoli become filled with protein-rich edema fluid and resident macrophages (a type of white blood cell) secrete pro-inflammatory proteins and cytokines [e.g., interleukin-8 (IL-8)], which recruit the innate immune cells (primarily neutrophils). Neutrophils adhere to the endothelial lining of the vessels and roll on this lining until they migrate through the alveolar-capillary membrane into the airspace, thereby damaging it. Neutrophil adherence to the endothelial wall can be measured via markers of endothelial injury such as soluble intercellular adhesion molecule-1 (sICAM-1 and ICAM-1) (Jochen Grommes, 2011). Neutrophils are activated in the alveolar space, which release granula proteins and reactive oxygen species (ROS) into this space. These proteases and oxidants cause further epithelial and alveolar injury and subsequent formation of hyaline membranes. Neutrophil activity can be measured by the expression of enzymes and proteins released by activated neutrophils, such as elastase or myeloperoxidase (MPO) (Johnson and Matthay, 2010; Fanelli and Ranieri, 2015).

### Pulmonary Metabolism

**FIGURE 4:**
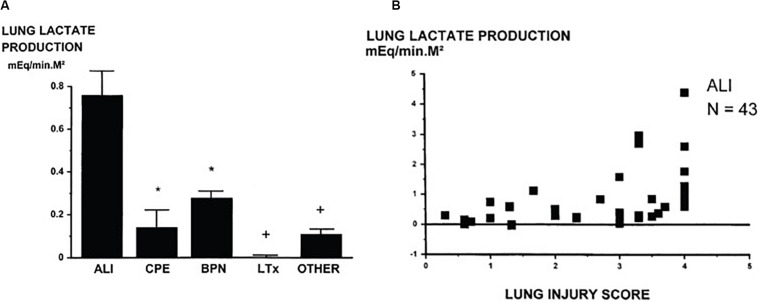
**(A)** Lung lactate production measured by the difference in the lactate concentration across the lungs (arteriovenous difference in lactate) in various groups of patients shows that lungs of ALI patients produce significant amounts of lactate. **(B)** Lung lactate production measured in 43 patients with acute injury showed that it is proportional to injury severity as determined by Murray’s lung injury score (Murray et al., 1988). (ALI, acute lung injury; CPE, acute pulmonary edema; BPN, bronchopneumonia; LTx, lung transplantation; Other, other types of respiratory failure, mEq/min.M^2^: mmoles per minute per square meter). Reprinted with permission of the American Thoracic Society. Copyright © 2019 American Thoracic Society from De Backer et al. (1997). The *American Journal of Respiratory and Critical Care Medicine* is an official journal of the American Thoracic Society. *p < 0.05 versus ALI, ^+^*p* < 0.01 versus ALI.

Healthy lung tissue predominantly relies on glucose utilization to sustain function, although its energy and metabolic needs are relatively modest compared to other organs such as the heart and liver (Fisher et al., 1974). Approximately 50% of the glucose utilized by the lung tissue converts to lactate (Fisher, 1984). The lungs typically maintain the glycolytic intermediary balance (lactate-to-pyruvate ratio) in the blood by utilizing the excess blood lactate, which results in a negligible difference in transpulmonary lactate concentration (Johnson, 2011). However, this function of the lung is compromised in many lung pathologies – especially in ARDS/ALI (Iscra et al., 2002). Figure 4 demonstrates lung lactate production measured by the difference in the lactate concentration across the lungs (arteriovenous difference in lactate) in 122 patients with a variety of lung disorders (De Backer et al., 1997). Lungs of patients with ALI (*N* = 43) produce significantly more lactate than other pathologies. What is more is that lactate production in ALI patients was strongly correlated with lung injury score as shown in Figure 4B. The injury score is representative of the severity of opacities observed in chest radiographs and computed tomography images, as well as the severity of overall respiratory failure measured by P_a_O_2_/F_i_O_2_ ratio and loss of pulmonary compliance (Murray et al., 1988).

Although increased glycolysis and lactate production by the lung tissue may reflect the presence of hypoxia due to elevated anaerobic metabolism, several studies suggest that the elevated lactate production by the lungs in ARDS/ALI is primarily associated with increased lung inflammation and neutrophil activity, and can occur even in the absence of tissue hypoxia. This suggests that increased lactate concentration and lactate-to-pyruvate ratio in the lung tissue may be a surrogate for lung injury and inflammation (De Backer et al., 1997; Kellum et al., 1997; Iscra et al., 2002).

## Position Emission Tomography

Positron emission tomography (PET) is a molecular imaging technique that enables visualization of metabolic and molecular processes by using a radiolabeled analog of a substance to interrogate specific pathways.

### Principles

The radiolabeled analog is first synthesized at a cyclotron facility by bombarding a radioligands with accelerated protons to produce unstable radioactive isotopes e.g., ^18^F and ^11^C that are then used in a biosynthesizer unit to produce radio tracers. The tracer is then injected intravenously into the patient. The nucleolus of the radiolabeled atom then undergoes β radioactive decay, in which a positron is released and travels for a short distance in the tissue (< 1 mm) before colliding with an electron to produce two γ-ray photons that travel in opposite directions (Pauwels et al., 2000), which are detected using a ring-shaped array of sensor around the patient. By resolving the time difference between the arrival of photons at sensors in the opposite direction, using the time-of-flight algorithm, 2D or 3D images can be tomographically generated (Gambhir et al., 2001). Data acquisition is often performed over a period of 30–60 min, providing temporal information as well. The raw data obtained from the scanner can be converted to markers of metabolic activity using timed-blood sampling to measure overall radioactivity combined with kinetic approaches that fit temporal changes of data to multi-compartmental models to decompose relative contributions of signal intensity from solid organs, blood and extracellular matrix (Chen et al., 2017).

PET scanners do not obtain anatomical information and thus are often combined with CT scanners (PET/CT scanners) to overlay the functional information on the anatomical images.

### Insights and Contributions

The most commonly used tracer for PET imaging is [^18^F]-fluorodeoxyglucose (^18^F-FDG). ^18^F-FDG is glucose analog and is similarly transported into the cell by glucose transporter 1 (GLUT-1) and subsequently phosphorylated. ^18^F-FDG cannot progress through the Krebs cycle and thus remains trapped in cells. Therefore, it can specifically be used to assess glucose uptake as a surrogate for overall glycolytic activity.

While ^18^F-FDG-PET is routinely used in neuro-radiology (Gambhir et al., 2001) and oncology (Pastorino, 2010; Heusch et al., 2014; Miles et al., 2018), it is not clinically used for the management of ARDS/ALI. However, several studies have demonstrated its capability as a powerful molecular imaging tool to delineate regions in the injured lungs with elevated glycolysis. As activated neutrophils are largely responsible for the uptake of glucose, elevated glycolysis can be used as a surrogate for inflammation. This has been validated in other studies that used autoradiography of the lung tissue in animals after administration of ^18^F-FDG showing that radioactivity was localized to neutrophils in the lung tissue.

Neutrophil recruitment and activation are heightened in ARDS, leading to elevated glycolysis, which can be regionally measured using ^18^F-FDG-PET. The ability of this molecular imaging technique to regionally highlight alterations in metabolic activity has made ^18^F-FDG-PET an invaluable research tool to non-invasively and quantitatively assess the severity lung inflammation. ^18^F-FDG-PET has been employed in both animal models of lung injury (de Prost et al., 2014) and in preclinical studies (Chen et al., 2006b; Bellani et al., 2012), and has provided valuable insight about the progression of lung injury (de Prost et al., 2014) as well as the importance of inflammation in patient outcome (Bellani et al., 2009, 2012).

In a preclinical study by de Prost et al. the authors assessed the impact of ventilation strategy on distribution and progression of lung inflammation using ^18^F FDG-PET (de Prost et al., 2013) in an endotoxemia model of lung injury in sheep. They found that primary inflammatory injury progressed rapidly in sheep ventilated with a non-protective strategy, while the inflammation was contained in animals ventilated with protective ventilation. Furthermore, the study demonstrated that metabolic activity was significantly higher in the dependent regions of the lungs.

**FIGURE 5:**
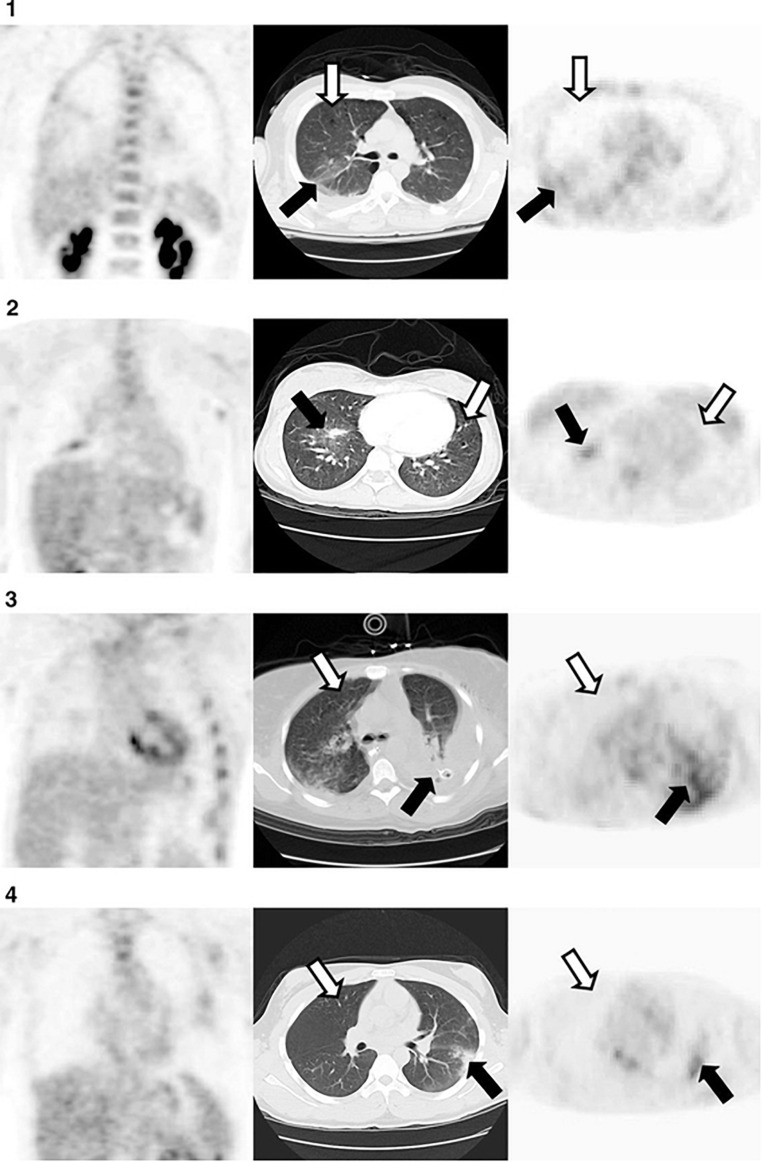
Representative axial computed tomography (CT) in the middle panel, and coronal (left) and axial (right) [18F] fluorodeoxyglucose (18F-FDG) positron emission tomography (PET) at the obtained from 4 patients, 72 hours after diagnosis with acute lung injury. Moderate uptake of FDG was observed in non/poorly aerated regions (black arrows). In contrast, uptake of FDG was low in normally aerated lung (white arrow). Reproduced with permission from Rodrigues et al. (2008).

In patients, ^18^F FDG-PET has been shown to be capable of localizing areas with higher neutrophilic activity the lung tissue as well. Chen et al. (2006b) showed that glucose uptake was significantly elevated in lungs of human subjects 24 h after instillation of 1–4 ng/kg endotoxin in airways using bronchoscopy. In a different study, Rodrigues et al. demonstrated the use of ^18^F-FDG-PET at early stages of ARDS (1–3 days after admission) to predict outcome (Figure 5); patients with significantly higher glycolysis in the lung tissue hard poorer outcome that others (Rodrigues et al., 2008). Bellani et al. (2009, 2011) showed the use of ^18^F FDG-PET to investigate the regional distribution of inflammatory activity in lungs of mechanically ventilated ARDS patients. Their work suggests that active inflammation is localized to non-dependent regions of the lungs at early stages. However, the inflammation spreads throughout the entire lung after days of ventilation. This data is supported by older work using localized biopsy that demonstrated similar destitution of inflammation (Papazian et al., 1998).

^18^F-FDG-PET have been used to localize inflammatory activity in other lung pathologies as well. Chen et al. (2006a) showed elevated FDG uptake in lungs of patients suffering from cystic fibrosis (CF) compared to healthy subjects (Chen et al., 2006a). The uptake rate correlated strongly with the number of neutrophils present in the bronchoalveolar lavage. What was more interesting was that the glucose uptake rate was especially higher in patients with rapidly declining pulmonary function. In a different study, the authors assessed FDG uptake in lungs of patients with chronic obstructive pulmonary disease (COPD) patients with and without chronic bronchitis (Scherer and Chen, 2016). They showed increased average uptake of FDG in lungs with patients with chronic bronchitis. The CT scans of the same patients also showed more heterogeneously distributed emphysema.

Although these studies suggest a strong link between neutrophilic inflammation and increased FDG uptake, other inflammatory cells such as macrophages and eosinophils are also capable of accumulating FDG (Scherer and Chen, 2016). Nevertheless, FDG uptake can represent a measure of overall inflammatory response in the lungs. Given that inflammation and inflammatory burden is associated with decline in lung function, disease severity and lung tissue destruction in many lung pathologies, ^18^F FDG-PET be used to classify patients with various disease severities to predict and assess response to treatments in these patients (Scherer and Chen, 2016).

There are a number of other less commonly used PET tracers that can provide more specific information about the inflammatory process. For instance, ^68^Ga-citrate has been shown to bind to the lactoferrin within the neutrophil, therefore localizing specifically neutrophilic inflammation (Scherer and Chen, 2016). 18F-nitric oxide synthase (18F-NOS) can assess expression of nitric oxide synthase in lung epithelium, which has been shown to be elevated in patients with progressive asthma, COPD, ARDS, and emphysema (Huang et al., 2015). Lastly, Summers et al. (2014) administered a bolus of ^111^Indium-tropolonate-labeled neutrophils to healthy subjects and ARDS patients and showed increased retention of neutrophils in and their delayed clearance in the ARDS patients.

### Challenges and Limitations

The first principal limitations for clinical use of PET imaging is the cost of preparing the radiolabeled compound, which is done at a cyclotron facility followed by an on-site chemical synthesis apparatus to produce the final compound. Such facilities are expensive to maintain and thus are available only at a few universities and hospitals. Therefore, radio tracers that have a long-half life, such as ^18^F-FDG (109.8 min) are often produced remotely at a cyclotron facility and transported to near-by locations. Since samples are radioactive, they need to be delivered via specially licensed road transport, or, for longer distances, via dedicated small commercial jet services, thereby making the scans costly. Another limitation is the long scan time (10–50 min) (Cereda et al., 2019) that is can be difficult for critically ill patients or patients with lung injury. Additionally, PET tracers expose patients to ionizing radiation, which limits use of this technique to monitor patient’s response to interventions through repeated measurements.

Another potential disadvantage of ^18^F-FDG-PET is that while it enables examining abnormalities in the uptake of the glucose analog fluorodeoxyglucose, it is unable to reveal changes in downstream metabolism as it cannot progress through the Kerbs cycle. Such information may be crucial to the evolution of inflammation and injury (Fisher and Dodia, 1984; De Backer et al., 1997). Lastly, given the long half-life of ^18^F-FDG-PET, the radioactivity of the probe from a single injection can last for hours, which limits the possibility of repeated scans as frequently as needed. This is especially important in small animal research, where lung injury progression occurs on a time scale that is significantly shorter than in human patients.

## Hyperpolarized ^13^C Magnetic Resonance Spectroscopic Imaging

Hyperpolarized ^13^C magnetic resonance spectroscopic imaging (MRSI) is a non-invasive emerging modality that enables delineation of different compounds via their distinct chemical shift, thereby making it suitable to assess flux critical branching points in metabolic pathways.

### Principles

^13^C MRSI enables study of metabolic flux in the tissue due to its unique ability to distinguish metabolites through their distinct resonance frequencies (chemical shifts). Due to low natural abundance of ^13^C nuclei, the MRI scan is performed after administration of an exogenous non-radioactive ^13^C-labeled compound. Subsequently, spectroscopic imaging methods can be used to highlight changes in cellularity and metabolic pathways. Although this method has been shown to be insightful for tumor and neuroimaging it is limited as it requires a long scan time due to low intrinsic nuclear spin of the ^13^C nuclei (Kurhanewicz et al., 2011, 2019). To overcome these challenges the signal can be increase through nuclear hyperpolarization.

Hyperpolarization is a process to temporarily enhance the sensitivity of the MRI signal by over 10,000-fold over conventional MRI (Kurhanewicz et al., 2011). Hyperpolarization of the ^13^C nuclei is typically achieved through a process called dynamic nuclear polarization (DNP), which transfers spin alignment from the sparse unpaired electrons of an electron paramagnetic agent (EPA) to the adjacent ^13^C labeled nuclei using resonant microwave irradiation at high magnetic fields (∼3.3T) at ∼1 K temperature (Ardenkjaer-Larsen et al., 2003). Once the sample reaches the desired polarization level (usually within 1–3 h), it is dissolved rapidly using a hot isotonic buffer to yield a highly polarized neutralized solution, which is then administered intravenously to the subject.

Upon injection, spectroscopic imaging must be carried out quickly and efficiently for two reasons; first, given the short life-time (T_1_ relaxation time constant) of the probes (10–120 s depending on the probe), the data must be acquired quickly. Second, in conventional MRI 2D or 3D images are acquired, whereas HP ^13^C MRSI requires data acquisition in the spectral dimension as well, which adds further complexity to the criteria for pulse sequence development. What is more is that any RF excitation causes additional irreversible signal loss. Several imaging and spectroscopic pulse sequences have been developed to address these challenges by limiting the number of excitations and exploiting the long T_2_, and T_2_^∗^ relaxation times of ^13^C species in many organs (Yen et al., 2009), as well as ^13^C species’ large range of the chemical shift (Kurhanewicz et al., 2011).

### Insights and Contributions

**FIGURE 6:**
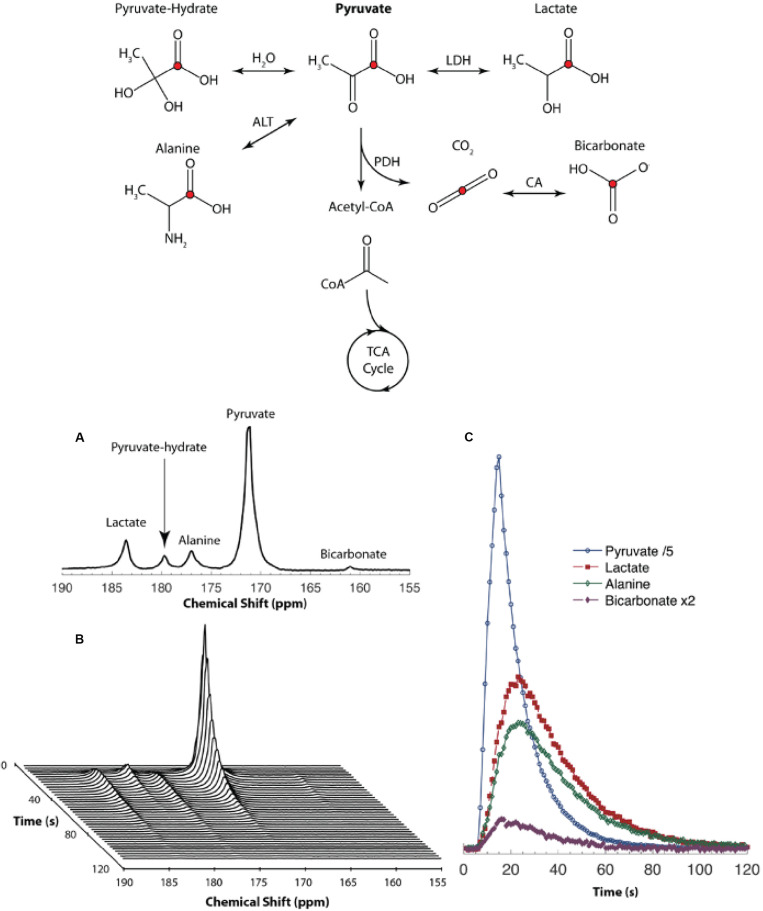
**(Top)** Metabolites and their biochemical pathways that can be interrogated using [1-^13^C] pyruvate. MRI. The red circle indicates atoms with ^13^C labeled nuclei. The fate of the pyruvate beyond Acetyl-CoA into the Tricarboxylic Acid (TCA) cycle cannot be probed using [1-^13^C] pyruvate as the ^13^C labeled nucleolus remains on the ^13^CO_2_ molecule. Cofactors are not shown in this diagram for simplicity (LDH, lactate dehydrogenase; ALT, alanine transaminase; PDH, pyruvate dehydrogenase; CA, carbonic anhydrase). **(Bottom) (A)** NMR spectrum obtained from a mouse after administration of hyperpolarized [1-^13^C] pyruvate via the tail-vein. **(B)** Spectra obtained every second shows how different peaks vary over time. **(C)** Area under each peak depicted as a function of time to represent the relative concentration of each peak. Reproduced with permission from Pourfathi (2019).

The most widely used hyperpolarized DNP probe is [1-^13^C] pyruvate, a small and highly soluble molecule with high polarizability (up to 60% polarization reported) and a long T_1_ relaxation time constant (40–60s). Because pyruvate is at a central branching point in several key metabolic pathways in cancer and inflammatory diseases, it be used to study a wide variety of metabolic perturbations in tissues and presents unique opportunities to characterize metabolic flux in various metabolic, Therefore [1-^13^C] pyruvate is perhaps the most attractive HP ^13^C imaging probe to date (Figure 6).

**FIGURE 7:**
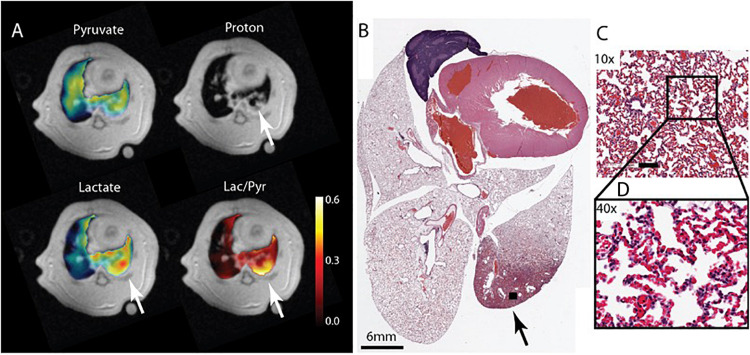
**(A)** Pyruvate, lactate and lactate -to-pyruvate segmented maps overlaid on their corresponding proton image of a ZEEP rat 4 h after the acid instillation shows injury to the posterior right lung marked by increased intensity in the proton image (white arrow). The metabolite maps show increased lactate signal intensity and lactate-to-pyruvate ratio colocalized with the injured area. **(B)** Hematoxylin and Eosin (H&E) axial slide of the whole lung clearly shows damaged lung tissue in the same area (black arrow). Magnified images taken from the injured area (black box) with **(C)** 10× and **(D)** 40× magnifications show severe damage and inflammatory infiltrates in the tissue. The bar in **(C)** is 100 μm. Reproduced with permission from Pourfathi (2019).

HP [1-^13^C] pyruvate has been used extensively to study metabolic alterations in heart (Lau et al., 2010), liver (Lee et al., 2013), kidneys (Laustsen et al., 2015), and tumors (Day et al., 2007) in intact animals and has more recently been used in a number of human studies (Nelson et al., 2013; Cunningham et al., 2016; Park et al., 2018). Nevertheless, given that the lungs receive the full blood supply during each circulation, and therefore, play a major role in maintaining body’s homeostasis and whole body’s metabolic status, HP ^13^C MRSI can be used as valuable tool for evaluating lung metabolism and pathology. In the context of lung injury, HP [1-^13^C] pyruvate MRSI can be used to regionally measure lactate-to-pyruvate ratio as a surrogate for lactate labeling, which is significantly elevated in inflamed lungs (Figure 7; Pourfathi et al., 2018; Pourfathi, 2019).

In an *ex-vivo* perfused lung study with an experimental model of bleomycin-induced lung inflammation in rats, Shaghaghi et al. (2014) showed a significantly increased rate of hyperpolarized lactate labeling after injection of hyperpolarized [1-^13^C] pyruvate in inflamed lungs. The lactate-to-pyruvate ratio declined but remained higher than healthy lungs even after fibrotic remodeling of the tissue at later stages of the injury. The study also showed a strong correlation between the lactate-to-pyruvate ratio and neutrophil scores derived from histological assessment of the lung samples. Interestingly there was no correlation between the macrophage count and the lactate signal suggesting that the primary source of the lactate is from neutrophils.

This technique has also been used for in-vivo imaging in small animals; Thind et al. (2012) showed elevated HP lactate-to-pyruvate ratio thus lactate labeling in irradiated lungs after radiation induced lung injury (RILI) compared to healthy animals. The authors showed a strong correlation between the lactate-to-pyruvate ratio and inflammatory markers measured from bronchoalveolar lavage. Pourfathi et al., showed that in an experimental model of aspiration pneumonia, HP lactate-to-pyruvate ratio was significantly elevated in mildly injured rats that received (intratracheally) a low volume of hydrochloric acid (HCl). Interestingly, the authors report a decline in the lactate-to-pyruvate ratio in more severely injured rats that received a larger volume of HCl. The authors showed that the although the lactate signal appeared to increase in the posterior regions of the injured lungs, the skewed measurement was in fact caused by a dramatic increase in the blood volume in the injured lungs.

Another study with HP [1-^13^C] pyruvate MRSI by Pourfathi et al. showed that this technique can be used to mechanisms of injury progression by secondary ventilator-induced lung injury; the authors assessed the impact of recruiting atelectasis on the trajectory of lung injury and inflammation in ventilated rats with primary aspiration pneumonitis, and reported that positive end-expiratory pressure (PEEP) and recruitment contains regional pulmonary lactate production and inflammation. The study supported a direct relationship between pulmonary inflammation and increased HP lactate-to-pyruvate ratio, consistent with the link between increased glycolysis caused by recruitment and activation of neutrophils as part of an innate inflammatory cascade, that was proposed by previous FDG-PET studies (Jones et al., 1994), and showed a strong correlation between the lactate-to-pyruvate and inflammatory markers of neutrophilic activity (MPO) and adherence (ICAM-1).

Finally, Siddiqui et al. showed that HP [1-^13^C] pyruvate MRI has the potential to be used as predictor for lung rejection. In a direct comparison between this technique and microCT in a lung allograft rejection rat model, the authors observed elevated lactate-to-pyruvate ratio prior to observing features in the microCT that are indicative of rejection. The authors also showed a strong relationship between the presence of markers of adaptive immunity CD4+ and CD8+, and the elevated lactate-to-pyruvate ratio in the transplanted lung (Siddiqui et al., 2019).

The preliminary results of these studies demonstrates the potential of HP [1-^13^C] pyruvate MRSI to detect elevated pulmonary lactate-to-pyruvate ratio. This imaging marker can serve as a surrogate to regionally assess increased glycolysis and subsequent lactate production by injured lungs as a result of inflammation (Pourfathi et al., 2017). While lactate mapping can be a marker of neutrophilic infiltration, it may be used to assess other biological changes in the lung related to lung injury that promote lactate production as well, such as fibrosis (Kottmann et al., 2012). The ability to interrogate alterations in critical downstream metabolic pathways such as glycolysis, may enable non-invasive and. frequent assessment of patients’ response to therapies early after treatment. This opens up opportunities to assess the response of various therapeutic apaches to select optimal strategy to attenuate lung injury and its consequences in the early stages (Shankar-Hari and McAuley, 2017).

### Challenges and Limitations

The most critical limitation of HP ^13^C MRSI is the very short lifetime of the hyperpolarization that limits the available “window-of-opportunity” to acquire data. Another limitation is the need for a clinical hyperpolarizer that is currently available at around 30 sites across the world. Unlike PET tracers, HP ^13^C agents cannot be produced remotely and delivered to the site-of-interest given that the lifetime of the hyperpolarization is significantly shorter than that of the half-life of PET tracers. Therefore, clinical dissemination of this HP ^13^C MRSI technology requires a polarizer at every site.

Other technical limitations of this technology for lung imaging in clinical studies are the field inhomogeneity in the lung tissue causing rapid spin dephasing at air-tissue interfaces and lung’s overall low tissue density. This difficulty is further exacerbated in the case of metabolic imaging by lung’s modest overall energy needs. These challenges limit both signal-to-noise ratio (SNR) and the suitability of rapid pulse sequences that are useful for imaging other organs. While many studies suggest that these challenges can be addressed (Pourfathi et al., 2017, 2018) the clinical utility of ^13^C HP MRSI in critically ill patients raises safety concerns primarily because of the need to use MRI–compatible monitoring and ventilation equipment.

Lastly, quantification of the absolute concentration of metabolites using HP ^13^C MRSI is non-trivial, as the absolute signal level is subject to variability due to polarization level and physiological conditions (Pourfathi et al., 2017). Although the use of hyperpolarized lactate-to-pyruvate ratio as a surrogate for endogenous lactate concentration (De Backer et al., 1997) and glycolysis (Siddiqui et al., 2016) can mitigate this variability, it can still be subject to bias caused by excess fluid in the extracellular space resulting from capillary bed leakage (Musch et al., 2007); the presence of such excess fluid can increase pyruvate concentration or limit its uptake, thereby reducing the lactate-to-pyruvate ratio and the sensitivity of the method (Pourfathi et al., 2017). PET imaging studies have addressed similar challenges by using compartment models to pinpoint the local source of signal (Chen et al., 2017). In the case of MRI, this bias may be corrected for by using rapid MRI pulse sequences that allow the acquisition of multiple images to characterize metabolic flux in various tissues (Siddiqui et al., 2016).

## Future Development

The pathophysiology of lung injury is complicated and entails changes in lung anatomy that arise from alterations at the cellular level and that compromise lung function. The molecular imaging techniques discussed here focus on the cellular changes which precede changes in the lung anatomy and function, thereby providing tools to detect inflammatory injury early and assess early response to treatment. Therefore, molecular imaging techniques may provide additional context to currently clinical tools to improve diagnostics and therapeutic approaches. Nevertheless, there are opportunities for improvement and further dissemination of either techniques to assess lung injury in a clinical setting.

Current research on PET imaging entails development of new hardware and analytical tools and algorithms, to improve spatial and temporal resolution. PET is often coupled with CT to capture an anatomical overlay of the metabolic maps. However, recent advancements in PET and MRI hardware technology and MRI pulse sequence development have created opportunities to combine PET and MRI together, that can potentially limit the ionizing radiation received by the patient. Additionally, in the case of lung injury, given the heterogeneity of tissue types with various disease, i.e., absence of solid tissue in healthy lungs and presence of edema or alveolar thickening in injured lungs, additional research is being performed to improve quantification of substrate uptake and flux (Chen et al., 2017).

Another potential area for future development is the use of deep learning methods to find spatial patterns that enables better classification of disease categories. While several studies have employed deep learning algorithms to classify CT images and to predict outcome (Merkow et al., 2017), its use has been much more limited with PET imaging (Singh et al., 2017). Nevertheless, addition of PET data to CT and other clinical markers can provide critical information about the severity of injury and its subsequent progression. However, a major limitation at this time is the absence of a large dataset of PET images obtained from the lungs of ARDS patients.

Hyperpolarized ^13^C MRI technology is at its infancy, yet it is showing tremendous potential to characterize branching points of the metabolic pathways in the presence of diseases. One major advantage of this technology is that it can potentially be coupled with other informative MRI methods, such HP ^129^Xe MRI. The latter is a non-invasive MRI technique that is capable of regionally quantifying lung function by measuring ventilation, oxygen uptake and apparent diffusion of gas in the alveolar airspace (Ruppert, 2014), thereby providing additional insight. Combined together, HP ^129^Xe and ^13^C MRI can obtain spatially correlated measurements of metabolism and function to provide a comprehensive insight into the pathology of the diseased lungs. Lastly, there has been significant progress in constructing inexpensive commercial and portable MRI machines. This can potentially make the use of MR-based molecular imaging methods to assess inflammation more accessible. This is particular important for imaging critically ill patients in the ICUs, where patient transport to the scanner is a major challenge. Despite the advantages, HP ^13^C MRI, generally suffers from hyperpolarization lifetime and challenges associated with lung MRI. While the use of T_2_-based pulse sequences may address some of these challenges (Pourfathi, 2019), there are several considerations that are required to address limitations with data acquisition and quantification (Yen et al., 2009).

Lastly, both methods exploit elevated glycolysis in activated inflammatory cells to localize pulmonary inflammation. As previously stated, while this provides a measure of integrated inflammatory activity in the lungs, it does not specify the type of cells present in the tissue. Future development for PET imaging can entail development of other substrates, similar to what was discussed earlier, to more specifically characterize the type of inflammation in the tissue. Moreover, additional novel tracers that are functionalized ligands that can bind to specific receptors may be synthesized to provide additional insight into the immune-pathogenesis of the disease (Kim et al., 2017).

Developing specific substrates for HP ^13^C MRI will be significantly more challenging that PET. This is because the life-time of hyperpolarization shortens significantly for larger and more complex molecules that can provide more specific information, thereby making imaging impossible. However, a number of substrates may be potentially useful; [6-^13^C] arginine has shown to be capable of reliably detecting the presence of myeloid-derived suppressor cells in bone-marrow (Najac et al., 2016). This substrate may be useful to study asthma as arginase, the enzyme that produces arginine, has shown to have a higher expression after allergen challenge in samples derived from asthmatic patients than healthy subjects (Arginine metabolism: enzymology, nutrition, and clinical significance. Proceedings of a symposium dedicated to the memory of Vernon R. Young. April 5-6, 2004; Bermuda, 2004). Another potentially useful substrate that may be polarizable is [1-^13^C] proline, which may be used to assess presence of fibrosis at later stages of lung injury (Wallace et al., 2002).

## Summary

In this article, we briefly discussed the critical role of lung inflammation in the outcome of patients with lung injury. We then provided an overview of two novel molecular imaging tools to regionally assess lung inflammation in the context of lung injury. Both exploit the elevated glycolysis and energy demand of activated immune cells that are present in the inflamed lung tissue; ^18^FDG-PET characterizes the uptake of glucose into the cell and its utilization, while HP ^13^C MRI quantifies the conversion of pyruvate to lactate thereby characterizing the fate of the glucose. Both techniques are valuable and can and can be used to assess lung inflammation or can combined together to provide a complementary picture of lungs bioenergetics. Such information could ultimately provide additional insight for clinical diagnosis and management of lung injury and ARDS and its trajectory. However, there are several technical challenges associated with either technique that requires to be addressed before their dissemination and ultimate clinical utility.

## Author Contributions

MP, SK, SC, and RR prepared the manuscript. All authors contributed to the article and approved the submitted version.

## Conflict of Interest

The authors declare that the research was conducted in the absence of any commercial or financial relationships that could be construed as a potential conflict of interest.
